# Scalable InGaN nanowire µ-LEDs: paving the way for next-generation display technology

**DOI:** 10.1093/nsr/nwae306

**Published:** 2024-09-20

**Authors:** Vignesh Veeramuthu, Sung-Un Kim, Sang-Wook Lee, R Navamathavan, Bagavath Chandran, Dae-Young Um, Jeong-Kyun Oh, Min-Seok Lee, Yong-Ho Kim, Cheul-Ro Lee, Yong-Ho Ra

**Affiliations:** Division of Advanced Materials Engineering, College of Engineering, Research Center for Advanced Materials Development (RCAMD), Jeonbuk National University (JBNU), Jeonju 54896, South Korea; Division of Advanced Materials Engineering, College of Engineering, Research Center for Advanced Materials Development (RCAMD), Jeonbuk National University (JBNU), Jeonju 54896, South Korea; Division of Advanced Materials Engineering, College of Engineering, Research Center for Advanced Materials Development (RCAMD), Jeonbuk National University (JBNU), Jeonju 54896, South Korea; Division of Physics, School of Advanced Sciences, VIT University Chennai Campus, Chennai 600127, India; Division of Advanced Materials Engineering, College of Engineering, Research Center for Advanced Materials Development (RCAMD), Jeonbuk National University (JBNU), Jeonju 54896, South Korea; Division of Advanced Materials Engineering, College of Engineering, Research Center for Advanced Materials Development (RCAMD), Jeonbuk National University (JBNU), Jeonju 54896, South Korea; Division of Advanced Materials Engineering, College of Engineering, Research Center for Advanced Materials Development (RCAMD), Jeonbuk National University (JBNU), Jeonju 54896, South Korea; Division of Advanced Materials Engineering, College of Engineering, Research Center for Advanced Materials Development (RCAMD), Jeonbuk National University (JBNU), Jeonju 54896, South Korea; Division of Advanced Materials Engineering, College of Engineering, Research Center for Advanced Materials Development (RCAMD), Jeonbuk National University (JBNU), Jeonju 54896, South Korea; Division of Advanced Materials Engineering, College of Engineering, Research Center for Advanced Materials Development (RCAMD), Jeonbuk National University (JBNU), Jeonju 54896, South Korea; Division of Advanced Materials Engineering, College of Engineering, Research Center for Advanced Materials Development (RCAMD), Jeonbuk National University (JBNU), Jeonju 54896, South Korea

**Keywords:** GaN, multi-quantum well, nanowire, micro LED, display, AR/VR/MR

## Abstract

Ever-increasing demand for efficient optoelectronic devices with a small-footprinted on-chip light emitting diode has driven their expansion in self-emissive displays, from micro-electronic displays to large video walls. InGaN nanowires, with features like high electron mobility, tunable emission wavelengths, durability under high current densities, compact size, self-emission, long lifespan, low-power consumption, fast response, and impressive brightness, are emerging as the choice of micro-light emitting diodes (µLEDs). However, challenges persist in achieving high crystal quality and lattice-matching heterostructures due to composition tuning and bandgap issues on substrates with differing crystal structures and high lattice mismatches. Consequently, research is increasingly focused on scalable InGaN nanowire µLEDs representing a transformative advancement in display technology, particularly for next-generation applications such as virtual/augmented reality and high-speed optical interconnects. This study presents recent progress and critical challenges in the development of InGaN nanowire µLEDs, highlighting their performance and potential as the next-generation displays in consumer electronics.

## Teaser

This review explores cutting-edge advancements in InGaN nanowire micro-LEDs, poised to revolutionize display technology with unparalleled brightness, efficiency and color accuracy, paving the way for the future of ultra-compact next-generation displays.

## INTRODUCTION

Display platforms are crucial for enriching human-digital interactions, as apparently ∼80% of global information uses light-based sensory channels [1]. With rapid advancements in communication and computing miniaturization, there is an increasing demand for more immersive interactions with digital interfaces like internet of thing (IoT) devices. Historically, following the cathode ray tube (CRT) era, liquid crystal display (LCD) technology emerged in 2000, quickly establishing itself as a dominant force in the display market. However, as technology advanced and drawbacks of traditional LCDs, such as limited viewing angles and high energy consumption, became apparent, there arose a demand for more immersive experiences. This demand led to the emergence of technologies like organic light emitting diodes (OLEDs), mini-light emitting diodes (m-LEDs), and micro-light emitting diodes (µLEDs), which offer self-illuminating capabilities, thinner panels, and better energy efficiency [2,3]. Table 1 summarizes key performance metrics of µLEDs in comparison to LCD and OLED technologies. These innovations, with compact sizes within the sub-micron range and fewer optical components, depart from traditional backlit displays [4]. They enable various formats, from expansive indoor/outdoor displays to immersive full-color augmented reality (AR) goggles, unlocking a multitude of applications. Although OLEDs have advantages, their lifespan is shorter due to material susceptibility. Amidst these challenges, transformative innovations, relying on µLEDs and advanced designs, are gaining increased attention.

**Table 1.** Key performance matrix of LCD, OLED, QD-LED and Inorganic Micro-LED.


	Type			
Properties	LCD	OLED	QD-LED	µLED

Operating mechanism	Backlit	Self-emissive	Backlit/self-emissive	Self-emissive
Energy consumption	Medium	Medium	Low	Low
Pixel density	Up to 1000 PPI	Up to 2500 PPI	Up to 30 000 PPI	Up to 30 000 PPI
Brightness	<2000 cd m^–2^	<1000 cd m^–2^	>106 cd m^–2^	>106 cd m^–2^
Contrast ratio	5000:1	>10 000:1	>1 000 000:1	>1 000 000:1
Lifetime	30 000–60 000 h	<10 000 h	1000–10 000 h	>100 000 h
Environmental stability	High	Medium	High	High
Moisture sensitivity	No	Yes	Yes	No
Flexibility	Low	High	Medium	Medium
Pixel size	Min. 32 µm	Min. 18 µm	Min. sub-micrometer	Min. sub-micrometer
Compactness	Low	Medium	High	High
Toxicity	Low	Low	High	Low
Operation temperature	−20 to 80°C	−50 to 70°C	−100 to 120°C	−100 to 120°C
Response time	Low (ms)	Medium (µs)	Very High (ns)	Very High (ns)
Visibility under sunlight	Medium	Medium	High	High
Cost	Low	Low	High	High

**Figure 1.**
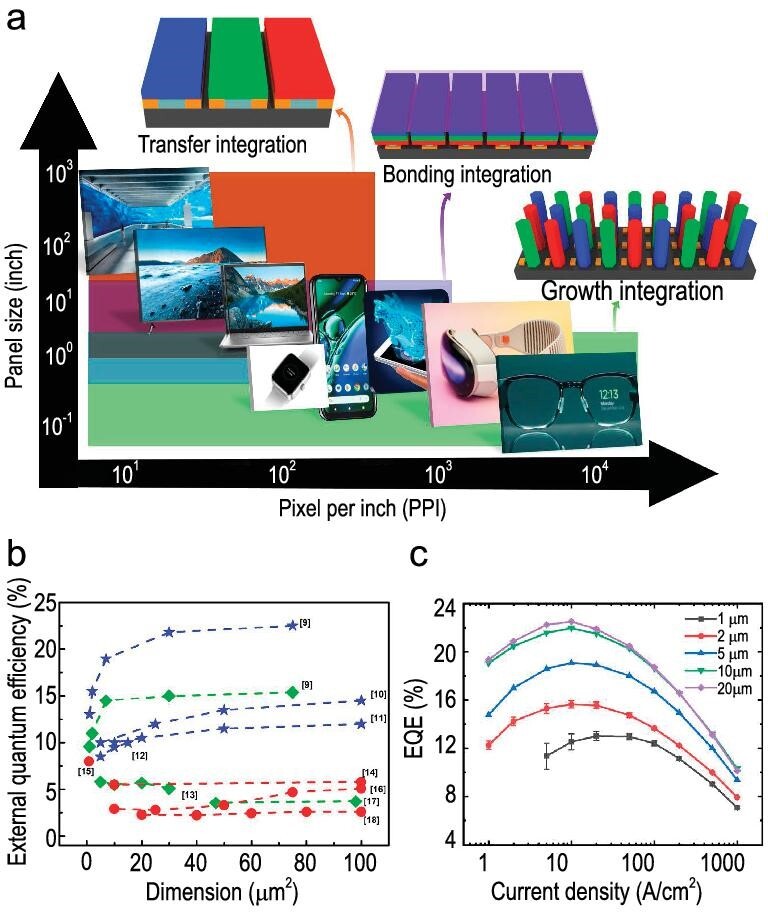
(a) Comparison of pixel density-dependent integration features of µLED displays with respect to panel size. Inset: various integration processes such as transfer, bonding, and monolithic integrations [8]; (b) presents a summary of recent publications that compile information regarding the active region dependence of external quantum efficiency (EQE) values in InGaN µLEDs [9–18]. (c) Size-dependent EQE values correspond to the injection current density. Adapted with permission from Ref. [9], Copyright 2022, AIP Publishing.

A µLED system is an array of submicron LEDs, each less than 100 μm in size, that are independently controllable and self-illuminating. Typically, these inorganic LEDs attain peak efficiencies at current densities of ∼0.1–100 A/cm², resulting in exceptional brightness with output luminance exceeding 100 000 cd/m²—essential for applications demanding high power [5]. As demand escalates, particularly for applications necessitating over 2000 pixels per inch (PPI) with ultra-high resolution, intense brightness, and vivid colors, conventional 2D flat-panel displays are being outpaced [6]. The transition to innovative 3D nanostructured configurations has become imperative to fabricate highly efficient and compact devices [7]. Various display driven lighting technologies are illustrated in Fig. 1a. This complexity arises from distinct growth and processing runs for each color, necessitating meticulous LED transfer for display realization. Additionally, their power consumption remains a substantial factor within electronic systems, particularly in portable or mobile devices.

The journey towards achieving commercial viability involves intricate optimization at the nanoscale, including reducing dislocation density, minimizing strain, and enhancing light extraction efficiency (LEE). InGaN nanowire (NW) LEDs show potential for high-resolution density and external quantum efficiency (EQE), achieving up to 58.5% internal quantum efficiency (IQE) with submicron diameters [19], utilizing their vertical structure to effectively guide light output as a waveguide [20]. The diameter-dependent emission wavelength of InGaN/GaN NW LEDs, driven by shorter lateral diffusion lengths of indium adatoms compared to GaN, allows for full-color displays through simple adjustment of nanowire diameter [21]. Typically, InGaN-NWs with minuscule lateral diameters can achieve high indium concentrations in their active regions with fewer indium adatoms, resulting in longer emission wavelengths. In contrast, larger diameter nanowires exhibit reduced incorporation of indium adatoms, thereby containing a smaller percentage of indium in their active regions, which leads to shorter emission wavelengths [22]. In particular, the InGaN/GaN dot-in-wire hexagonal LED stands out due to its diameter-dependent emission wavelength and excellent electrical performance. Figure 1b illustrates the recent advancements in the EQE of InGaN µLED, corresponding to emissions in the red (R), green (G), and blue (B) spectra. However, sub-wavelength nanowires pose challenges such as different angular radiation patterns in the far-field, causing noticeable angular color shifts due to varied waveguide modes. This marked reduction in efficiency under minus-scale presents a substantial hurdle in the path towards the extensive acceptance and commercial viability of µLED technology, see Fig. 1c. Recent studies by Y. Qian *et al*. [22] demonstrate that optimizing nanowire geometry can narrow the viewing cone to within ±20°, significantly enhancing LEE across RGB nanowire LEDs, while ensuring that this shift remains imperceptible to the human eye's just-noticeable difference.

In the landscape of display technologies the ascent of InGaN nanowire µLEDs marks a significant milestone in the evolution of display technology. With their potential to redefine visual experiences across various applications, these tiny but powerful LEDs are poised to illuminate the future of how we perceive and interact with digital information. Longer wavelength emissions necessitate a high indium concentration, leading to a primary issue of point lattice mismatch due to defect formation. To address these challenges, the concept of a photonic crystal engineering process, which intricately scores the generation, transport, and recombination of carriers, presents a promising solution to address current challenges and achieve enhanced excitonic effects [23–26]. This involves direct growth to alleviate challenges in realizing RGB emission µLEDs, particularly addressing efficiency concerns at longer wavelengths and in the red wavelength region also.

For large-scale displays, integrating discrete device dies is essential to create an extensive micro-LED array. Similarly, achieving a multicolor display requires the integration of red, green, and blue µLED units on a single substrate [27]. Various integration technologies, including transfer integration, bonding integration, and growth integration, are employed for µLED displays [28]. The development of the integration concept of excitonic µLEDs has been a straightforward approach to address challenges related to precision thresholding, time-consuming processes, and performance issues [8].

## MINIATURE PIXELIZATION CHALLENGES OF MICRO-LEDs

Micro-LEDs emerge as a transformative force, holding the promise of revitalizing industries through their adept integration of high-resolution fine pixel displays. These tiny powerhouses seamlessly bring together mono/multi-chromatic pixels on a single microchip, ushering in a distinctive style eagerly embraced by cutting-edge display technologies [29]. Leveraging these inherent advantages, RGB µLEDs ascend to prominence as luminosity beacons, captivating attention across a spectrum of applications—from dynamic displays to fashionable wearables, and from immersive augmented reality experiences with incredible brightness to cutting-edge virtual reality setups. This surge in interest drives the quest for efficient RGB emitters, an ambitious endeavor aimed at seamlessly integrating these technological advancements into practical, real-world applications [30,31]. As of large-area inorganic LEDs, InGaN and AlInGaP alloys have traditionally served as the primary materials for producing blue-green and yellow-red emissions in µLEDs, respectively. However, the challenge arises when addressing the need for small pixelation, as AlGaInP red LEDs face a significant obstacle—elevated leakage current across the junction temperature, leading to a rapid decline in efficiency. Additionally, AlGaInP-derived red LEDs encounter compatibility issues with InGaN-based blue/green LEDs. These issues primarily stem from disparities in light angular distribution and significant discrepancies within the operational voltage range of the devices, posing challenges in terms of material compatibility and efficiency maintenance [32]. Beyond this, color conversion technology, involving the integration of phosphors onto III–V semiconductors, encounter hurdles such as low efficiency, inconsistent color output due to cross-talk, and size limitations. Meanwhile, Quantum-dot-based color conversion technologies, though promising for µLED displays, introduce complexities and increased costs due to additional fabrication steps, along with concerns about saturation, degradation, and the toxicity associated with heavy metals (Pb and Cd [33]). Conversely, the spotlight has shifted towards the highly efficient InGaN-based red LEDs, which, boasting diminished size impact and seamless integration with InGaN-based blue/green counterparts, emerge as compelling contenders poised to replace AlGaInP-based red μLEDs for the realization of vibrant full-color displays.

The decrease in efficiency in µLEDs, known as the efficiency cliff, is primarily attributed to increased surface recombination as lateral dimensions decrease. Notably, in the intriguing process of adatom infusion into the InGaN region, blue emissions occur at a relatively lower range of 15%–17% indium content. For green light emission, ∼22% indium is needed, while achieving red illumination requires a significantly higher indium concentration. As the indium composition within the quantum wells intensifies, a captivating account unfolds—the quantum efficiency of InGaN LEDs embarks on a journey of decline, especially in the landscape of longer wavelength emissions [34,35]. Known as the ‘green gap,’ this contributes to the degradation of crystal quality and the occurrence of the quantum-confined Stark effect (QCSE) [36]. Typically, the demanding composition standards pose a significant hurdle, impacting overall efficiency due to lattice mismatch between the substrate and epilayers, as well as among the epilayers themselves, resulting in various structural defects like point defects [37], threading dislocations [38,39], stacking faults [40–42], trench defects [43–45], V-pits [39,46], and misfit dislocations [47,48] in InGaN LEDs. These structural defects not only affect crystal quality but also act as nonradiative centers, initiating the Shockley–Read–Hall nonradiative process. The necessity for a low growth temperature to prevent indium desorption creates a trade-off, compromising surface morphology, and leading to poor quality and elevated defect density [49,50]. Additionally, the immiscibility of InGaN alloys results in compositional fluctuations and phase separations, hindering the attainment of high-quality InGaN layers [51]. Meanwhile, power consumption and heat generation are interlinked issues, as excess heat can elevate junction temperatures, alter emission wavelength, and reduce the EQE of µLEDs. Addressing crystal quality concerns, lattice mismatch-induced compressive strain in the LED epitaxial structure poses challenges such as QCSE, compositional pulling effect, and obstacles to achieving long-wavelength emission in InGaN LEDs, along with universal device challenges like efficiency droop [52–58], carrier injection [59–62], and light extraction [63,64], emphasizing the need for comprehensive solutions to enhance their performance.

## EMERGENCE OF MONOLITHIC InGaN NANOWIRE MICRO-LEDs

Wurtzite-InGaN, with its distinctive polarization and robust QCSE, adeptly separates electrons and holes within GaN/InGaN quantum wells, which is crucial for driving LED light generation. However, their high density of dislocation due to the prevalent lattice mismatch in III-nitride quantum wells results in a significant QCSE, reducing the alignment of electron and hole wavefunctions and compromising luminescence quantum efficiency and lifetimes. Exploring the potential of GaN-based nanowires emerges as a compelling strategy to alleviate strain and confine dislocations at the nanowire base, owing to their substantial length-to-diameter aspect ratio—a pivotal advancement in LED technology. Fine-tuning in growth, including adjusting atmospheric conditions and regulating source composition, allows for the control of nanowire morphology and indium compositions in InGaN quantum wells, facilitating the production of emission wavelengths across the entire visible spectrum. Challenges persist in understanding coalescence processes and morphology evolution, particularly in fabricating high-quality InGaN quantum wells. In contrast to top-down etching methods with inherent etching damages, the bottom-up growth approach exhibits superior material quality across various substrates. This quality opens up possibilities for innovative devices, including single-photon sources and plasmonic layers. Despite historical discussions on the growth mechanisms of GaN nanowires, challenges persist in understanding coalescence processes and morphology evolution, particularly in the fabrication of high-quality InGaN quantum wells. Therefore, the emphasis has shifted towards achieving well-separated nanowires and ensuring a uniform distribution of indium, as these factors have become increasingly crucial for optimizing device performance in this intricate landscape.

**Figure 2:**
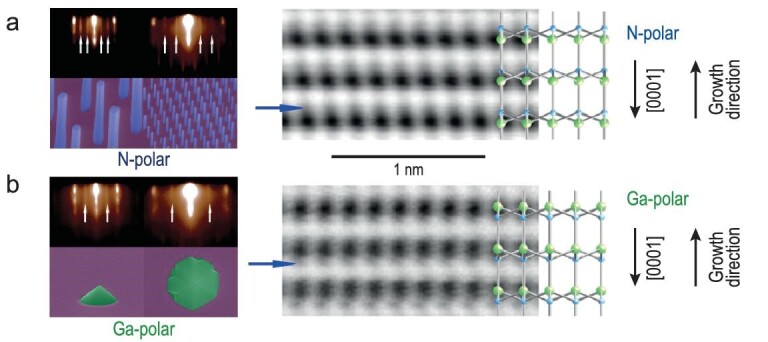
Illustration of the PFM phase/polarity and annular bright-field STEM lattice images of GaN, showcasing the (a) N-polarity and (b) Ga-polarity lattice arrangements of GaN. Adapted with permission from Ref. [66], Copyright 2016, American Chemical Society.

Polarity manipulation, surface treatment, and defect minimization are critical for enhancing crystal quality, reducing threading dislocations, and increasing light extraction efficiency in III-nitride materials. The termination of III-nitride surfaces by either metal (Ga/In) or nitrogen (N) atoms dictates whether the material is metal (Ga/In)-polar or N-polar [65]. This ‘crystal face polarity’ profoundly impacts growth processes and fabrication techniques, enabling precise control over band alignments, carrier transport properties, and light emission characteristics. Figure 2 illustrates RHEED and annular bright-field STEM lattice images of GaN, showcasing the (a) N-polarity and (b) Ga-polarity lattice arrangements of GaN [66]. Ga-polar nanowires are noted for their superior carrier mobility, reduced threading dislocation density, and enhanced light extraction efficiency compared to N-polar nanowires. However, larger diameter nanowires often exhibit interior voids and irregular facets. Recent advancements have shown that growing GaN nanowires on N-polar GaN substrates under metal-rich conditions results in void-free structures with smooth facets [65]. N-polar III-nitrides, grown at higher temperatures under N-rich epitaxy conditions, enhance p-type conduction efficiency by suppressing N vacancy-related defects and minimizing electron leakage, which is crucial for high-efficiency emission in the deep visible spectrum [67,68]. During the growth of (In, Ga)N along the N-polar direction, enhanced indium incorporation efficiency can be achieved due to the higher thermal dissociation limit of N-polar InN [69,70]. Additionally, N-polar GaN/(In\Al)GaN nanowires exhibit improved selectivity, vertical alignment, flat top surfaces, reduced lateral spreading, and enhanced vertical growth characteristics [65]. N-polar InGaN nanowires grown along the c-axis feature flat top surfaces, simplifying device fabrication and enhancing yield, making them suitable for high-power device applications [71,72]. Nonetheless, N-polar nanowires showcase benefits in mitigating polarization-induced electric fields and refining QCSE modulation, fostering superior color adjustability and efficiency mitigation against droop. Ongoing research into Ga and N polarity-controlled growth of InGaN nanowire µLEDs holds great promise. Although nonpolar growth theoretically offers advantages by eliminating polarization field drawbacks, significant technological challenges persist in achieving high luminescence efficiency [71]. In the complex domain of nanowire platforms, integrating Quantum Dots Nanoparticles (QDNPs) with high quantum yield or the capability to generate Localized Surface Plasmons (LSPs) utilizing noble metal nanoparticles (Au and Ag) is vital for efficient photon energy down-conversion, representing substantial progress in LED technology [73–75].

Exploring progress in InGaN µLEDs involves overcoming challenges like maintaining high IQE despite dislocation density, addressing ‘current droop’ reducing IQE at higher currents, and tackling efficiency decline in wavelengths beyond blue, known as the ‘green gap.’ Key factors include minimizing surface recombination-induced carrier losses linked to LED chip size and addressing current crowding causing efficiency droop in concentrated current regions.

## PROGRESS ON InGaN NANOWIRE MICRO-LEDs

Using a conventional top-down approach, III-nitride µLEDs undergo dimension reduction using standard photolithography and subsequent dry-etching processes. While dry etching has minimal effects on larger LEDs (>100 μm), its impact becomes significant for smaller µLEDs. Variations in the velocities of surface rearrangement are ascribed to the influence of convergence, leading to enhanced surface rearrangement during the plasma etching phase used to delineate device mesas. Unfortunately, this step leads to significant surface damage, causing crystal defects, dangling bonds, and the presence of impurities around the mesas. This poses a significant threat to high-resolution displays in carrier injection properties, especially in scenarios with a high surface area to volume ratio, potentially leading to higher power consumption and self-heating during operation. To address these challenges, such as surface passivation technique [76], including annealing, exposure to nitrogen plasma, wet chemical etching, and surface treatments, and pixelization methods with heavy ion implantation [77]. The plasma etches a kind of surface passivation that affects the p-doped layer, creating an N-deficient near-surface region due to plasma exposure. Point defects like these typically serve as compensators for Mg acceptors, resulting in a reduced abundance of free holes. This reduction can potentially influence the carrier transport characteristics of the device. However, a combination of thermal annealing and N_2_ plasma exposure enhances surface stoichiometry, aiding in device characteristic recovery. Hydrogen plasma treatment implies better EQE by 1.4 times in green InGaN-based µLEDs, deactivating Mg acceptors and mitigating charge carrier injection issues near surface defects. Immediate post-mesa etching surface treatments involve atomic layer deposition (ALD) for dielectric Al_2_O_3_ deposition or (NH_4_)_2_S treatment, effectively passivating surface states. Sol-gel SiO_2_ passivation achieves blue-emitting InGaN µLEDs with an EQE of ∼20.2%, proving over 2 times more effective than plasma-enhanced ALD-deposited SiO_2_. The low-temperature sol-gel approach minimizes the creation of additional surface or structural defects. Continuing with chemical treatment and sidewall passivation, wet etching, commonly employing KOH, is instrumental in eliminating leakage paths that arise during plasma etching. This process contributes to the restoration of device performance by selectively removing ∼50–60 nm of the semiconductor material. While these methods show improvements in peak EQE values and decreased dark current, their reliance on post-growth techniques raises concerns about achieving perfect sidewall passivation or defect-free pixelization. To effectively address the decline in efficiency associated with size-dependent factors, it is crucial to fundamentally diminish the impact of surface effects. Despite extensive studies, the efficiency of µLEDs produced through conventional top-down etching remains limited, particularly for green and red devices [13,78].

Amidst the intricate hurdles hindering µLEDs efficiency, integration, and long-wavelength operation, a captivating path emerges of nanostructures. These minuscule yet potent elements promise to surmount challenges and elevate micro-LEDs to unparalleled efficiency. Embracing nanostructures not only follows a trend of minuscule footprint, but also unlocks a range of solutions, paving the way for cutting-edge high-performance µLED technology. The paradigm shift in μLED technology focuses on refining defect-free epitaxial nanowires through techniques like MOCVD and MBE. These nanostructures, characterized by high-quality crystals and enhanced surface-to-volume ratios, play a pivotal role in indium integration and dopant incorporation. This enhances carrier recombination rates and LED injection efficiency, crucial for achieving full-color µLED displays. Additionally, integrating wide-bandgap AlGaN into InGaN structures forms a protective core-shell/sandwich configuration, mitigating surface recombination effects. The meticulous control over nanostructure dimensions, shape, morphology, and placement prior to epitaxy is facilitated by the structural pattern control growth process of SAE into both MBE and MOCVD. Growth and substrate conditions play a crucial role in determining morphology, significantly influencing the final outcome. For instance, research has shown that multi-color emission (RGB) can be achieved by varying the dimensions of InGaN/GaN nanowires through MBE-SAG, shedding light on size-dependent emission control properties and shadowing effect on composition configuration in an array. For instance, K. Kishino *et al*. [21] reported that the realization multi-color emission (RGB) had been achieved by varying the dimensions of InGaN/GaN nanowires through the use of MBE-SAG. This report provides insights into size-dependent emission control properties, and it highlights how the shadowing effect of epitaxy can influence the composition configuration in an array. Continuously, micron-scaled platelets, pyramids, and nanowires using SAE-MOCVD for µLEDs, are also investigated [79,80]. J. Bai *et al*. [81] showcased µLEDs featuring a remarkable 3.6 μm diameter and a close-fitting 2 μm lattice constant. Significantly, the researchers improved performance by integrating epitaxial lattice-matched distributed Bragg reflectors (DBRs) into the substrate-assisted epitaxy (SAE) process for µLEDs, resulting in an outstanding EQE of 9% at ∼500 nm [82]. Subsequently, their research progressed to InGaN nanowires utilizing a technique involving a microhole array into SAE at higher temperatures. This method facilitated the creation of structures with dimensions as small as 2 µm, demonstrating red µLEDs emitting at 642 nm with an impressive luminance of 3.5 × 10^7^ cd/m² and a peak external quantum efficiency of 1.75% [83]. Achieving full-color μLEDs involves advancing multi-color LEDs on a single wafer, enabled by selective area epitaxy (SAE) with patterned masks for precise control, reducing surface defect density of uniform array cultivation over large surfaces. By adjusting the metal flux ratios of indium and gallium, the resulting alloys composition can be finely tuned. Monolithic growth of multi-color devices is achieved through SAE with a patterned mask for precise control, reducing surface defect density despite high indium incorporation due to efficient strain relaxation advantages [21,84,85]. Furthermore, photonic nanoengineering crystals play a vital role in improving emission properties, including enhancing light extraction efficiency, emission directionality, and narrowing spectral output. These advancements extend the utility of nanostructure-based surface-emitting lasers as well.

Integration of p-conduction, typically facilitated by Mg-doped GaN, with InGaN heterostructures stands pivotal in bolstering the quantum efficiency of the active region, a vital facet for superior device performance. Mg doping goes beyond mere enhancement of electrical conductivity; it encompasses structural refinement, modulation of surface charge upon integration, and impacts on the photochemical properties of Mg-doped GaN nanowires. Ra *et al*. [86] demonstrated insights into the intricate mechanisms of Mg doping in p-GaN nanostructures and its correlation with emission efficiency, enabling precise control to improve electrical performance by fostering high-quality crystal structures and suppressing defects within p-GaN nanocrystals. Excessive Mg doping may introduce leakage currents due to Mg impurities, highlighting the need for a delicate balance to achieve optimal device performance.

## PIXEL INTEGRATION TECHNIQUES FOR MASS TRANSFER PROCESS

**Figure 3.**
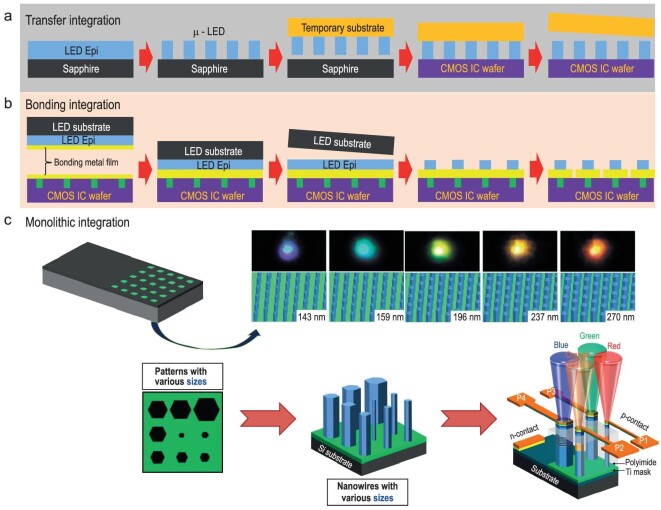
Schematic representation of integration technologies such as (a) transfer integration, (b) bonding integration, and (c) monolithic integration. Adapted with permission from Ref. [85], Copyright 2016, American Chemical Society.

Fabricating large, immersive screens involves assembling individual dies and configuring drivers to control emission, culminating in the development of fully operational µLED devices, sized <50 µm. Addressing the intricate challenges posed by the compact, finely-tuned pixels of µLED displays involves a multifaceted undertaking, particularly in terms of array arrangement and system integration. This integration can be categorized into three main facets: (a) transfer integration, (b) bonding integration, and (c) monolithic integration, see in Fig. 3 [8].

Transfer integration involves mass transfer, using a pick-and-place technique for seamlessly integrating µLEDs into pixel arrays with driving circuits, forming a 2D or 3D integration. Various tool-driven transfer methods and their characteristics such as stamping, laser selective-release transfer, electrostatic and electromagnetic pick-up transfers have been reported [87]. Fluidic assembly, utilizing gravity and capillary forces, has also been explored for mass transfer. After a successful transfer, the p-electrodes and n-electrodes of individual µLEDs are connected in specific arrangements, creating matrix-addressable driving patterns. However, there exists a demand for greater accuracy, cost-effectiveness, and time-saving platforms that current methods struggle to fulfill.

In the case of bonding integration, known as wafer bonding which seamlessly merges identical or diverse materials onto a single substrate, a fascinating avenue unfolds. The fusion of vertically stacked hybrid wafers in wafer bonding integration gave rise to an intriguing breakthrough—color-tunable LEDs. Liu *et al*. [88] showcased UV and RGB µLED arrays in 2013, progressing at 360 pixels per inch (PPI). A crescendo occurred in 2014 with blue µLED arrays featuring a 15 μm chip size and an impressive 1700 PPI [89]. A strategically implemented vertically oriented waveguide structure within the hybrid pixel played a crucial role in optimizing light extraction. The allure of integrated bonding resides in its adeptness at meticulously sculpting µLED dies directly onto the wafer using photolithography. This innovation, pioneered by Chun *et al*. [90] involved bonding GaN-based blue and AlGaInP-based yellow LED epitaxial layers, utilizing an ITO layer as an adhesion agent. Subsequently, the fusion of metal bonding and stacking epitaxy wafers, each emitting unique primary color wavelengths [91], led to the inception of a µLED array with a pixel pitch ranging from 5 to 10 μm. In 2011, a green µLED array with a dimension of 160 × 120 was fabricated utilizing indium bump flip-chip bonding technology [92]. In 2020, Wang *et al*. [82] demonstrated a green µLED array with a 3.6 μm chip size on a sapphire substrate, showcasing integrity preservation through bypassing mesa etching. For instance, in 2023, Hwang *et al*. [93] reported the successful wafer-scale alignment and integration of a µLED array using a mass transfer process via selective bonding, hinting at the tantalizing possibility of achieving minuscule pixel dimensions and remarkably high resolutions. However, achieving top-notch resolution in micro-LED displays proves challenging through the conventional method of device preparation preceding bonding due to precision alignment constraints. Conversely, fabricating the device post-bonding allows for potential pixel size reduction akin to the continual scaling down in CMOS processes, contingent upon lithography accuracy. Thus, prioritizing bonding-first fabrication techniques offers advantages for crafting high-resolution and high-throughput micro-LED displays. Nonetheless, ensuring stability in bonding materials and compatibility with subsequent procedures remains imperative. Despite the facilitation of high-resolution display preparation via wafer-bonding integration, the display area inherently remains bound by the epitaxial wafer's size. Consequently, wafer-bonding integration serves as a fitting choice for manufacturing diminutive wearable micro-displays, given its suitability for small-scale production.

In the dynamic landscape of silicon-based devices, there is a growing emphasis on achieving compact yet high-performing systems, leading to a significant shift towards monolithic integration methods, known as growth integration. This departure from conventional wire bonding approaches in packaging integration has directed attention towards wafer-level electrode interconnections, signaling a new era for Systems on a Chip (SoCs) that are both sleek and effective. Growth integration, characterized by the integration of material development and device manufacturing on a single wafer, represents a forefront of innovation. Direct integration of micro-LEDs through growth integration is recognized as crucial for achieving exceptional frequency and current density in a remarkably condensed form factor. Bypassing the mass transfer process not only achieves seamless emission in a compact size, but it also yields substantial economic and time-saving benefits. The continuous variation in indium content within InGaN/GaN LEDs results in a dynamic spectrum of emitted light, smoothly transitioning from blue to red [21,94–96]. This characteristic provides these LEDs with a distinct advantage, enabling seamless integration of flawless, multi-colored illumination. In 2010, H. Sekiguchi [21] demonstrated precise control over emission colors in InGaN/GaN nanowire array LEDs, achieving a range from vibrant blue to rich red. This achievement was enabled by integrating monolithic structures, where nanowires of varying diameters, ranging from 137 to 270 nm, were grown on a single wafer. As the diameter of the nanowires increased, intricate phenomena such as the beam shadow effect and distinct diffusion behaviors of Ga and In adatoms along the sidewalls emerged. This complex interaction led to a notable observation: larger nanowires exhibited reduced migration of Ga atoms towards their summits, resulting in a higher In composition within the InGaN wells. This increased In presence led to a narrower bandgap within the InGaN quantum well, consequently emitting light at longer wavelengths. Expanding on this approach, Kishino *et al*. [84] demonstrated the monolithic integration of green and orange InGaN/GaN nanowire array LEDs. Subsequently, the integration of 4-color LEDs (red, green, blue, and yellow) was demonstrated [96]. Exploiting the size-dependent emission tunable property, Ra *et al*. [85] demonstrated on-chip monolithic integration of ROGB micro-LEDs, leveraging the tunable emission of single nanowires array. However, challenges emerged in attaining precise control of In content due to lateral overgrowth of nanowires, necessitating a delicate balance.

**Figure 4.**
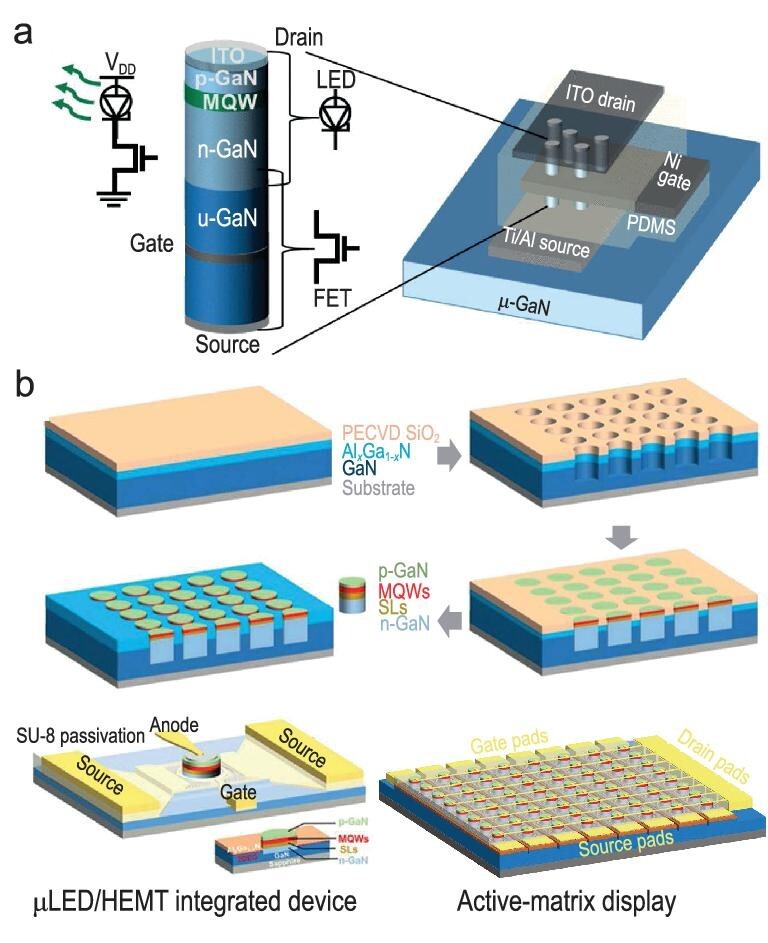
(a) Conceptual design of vertical GaN nanowire LEDs integrated with nanowire FETs (reproduced with permission from Ref. [99]), Copyright 2019, IEEE, while (b) depicts the process flow for fabricating µLED/HEMT hybrid wafers using selective area growth (SAG). The bottom left image shows the fabricated µLED/HEMT integrated device, and the bottom right image illustrates the configuration of an active-matrix display utilizing these integrated components. Reproduced with permission from Ref. [100], Copyright 2021 which licensed under CC-BY, American Chemical Society.

In advanced display and integrated electronic applications, integrating µLEDs with their driving transistors using complementary metal-oxide-semiconductor (CMOS)-backplane controlled active matrix (AM) micro-displays is crucial [97,98]. CMOS driver circuits in AM configurations, composed of p-MOS and n-MOS transistor arrays along with power and signal lines, ensure low power consumption and reliable transistor control based on external signals. Figure 4a illustrates a vertically stacked nanowire GaN gate-all-around (GAA) FET integrated at the base of a nanowire InGaN/GaN LED structure [99]. This configuration, achieved through top-down etching, positions a nanowire LED on top while the GaN-FET is fabricated using an undoped GaN (u-GaN) nanowire as the current channel. By stacking the GaN FET and LED vertically instead of laterally, the design bypasses the need for regrowth and metal interconnects, preserving the LED area. However, a significant challenge is the large leakage current in the GaN GAA FET, which can adversely affect grayscale control and power consumption in display applications. Later on, J. Bai *et al*. [82] demonstrated a direct epitaxial ultrasmall and ultrabright InGaN µLED/HEMT using a SiO_2_ mask pattern template, as shown in Fig. 4b. This led to the creation of ultrasmall, ultra-efficient, and ultra-compact green µLEDs with dimensions of 3.6 µm and an interpitch of 2 µm. Figure 4b depicts the typical selective epitaxial growth of an AlGaN/GaN heterojunction wafer by plasma-enhanced chemical vapor deposition using an SiO_2_ mask pattern. The selective epitaxial area was defined by lithography and dry etching, exposing the 2DEG at the AlGaN/GaN interface on the etching sidewall. When the micro-LED system is selectively grown, the n-GaN layer is electrically connected to the 2DEG. After removing the SiO_2_ mask, a hybrid wafer containing micro-LED and HEMT was obtained. Subsequently, planar processes such as photolithography and etching were used to fabricate integrated devices. The ring-shaped gate electrodes effectively control the current flow of the HEMT device and drive the corresponding micro-LED in series (see Fig. 4b). After matrix interconnection, an 8 × 8 micro-LED microdisplay was fabricated, with the micro-LED size and pitch being 20 µm and 25 µm, respectively (Fig. 4b). This growth integration scheme reduces the reliance on heterogeneous integration, making it possible to produce micro-LEDs and their compatible components on the same GaN platform [81,101]. However, GaN-based transistors as drivers facilitate the growth and integration of micro-LED displays in terms of material compatibility. Optimizing with enhancement-mode transistors, which typically operate in an off-state, is preferred to reduce energy consumption and enhance circuit safety. Forming ohmic contacts in GaN transistors requires heat treatment above 800°C, surpassing the thermal tolerance of LED devices and affecting their performance. Recently, Xing *et al*. [102] demonstrated monolithic integration of GaN-based LEDs and vertical enhancement-mode GaN MOSFETs by selectively regrowing a p-GaN and n-GaN bilayer atop an LED structure. This approach enabled normally-off operation of the GaN transistor but increased on-resistance and power consumption due to the p–n junction barrier created. Meanwhile, LG display unit [103] has demonstrated an advanced monolithic µLED technology that combines nitride-based LEDs with silicon thin-film transistors (TFTs) on a gallium-nitride-on-silicon substrate for AM displays. This process involves creating MOS TFTs on the silicon surface revealed after dry etching of the LED epitaxial layer. This innovative integration produced a 150 PPI, 0.6-inch monolithic display featuring a 60 × 60-pixel array on a single substrate. This design leverages Si-based MOSFETs and the well-established Si CMOS process, driving forward the evolution of micro-LED display technologies.

Monolithically integrating micro-LEDs directly grown on a substrate embedded with driving transistors offers significant advantages, enhancing power efficiency by minimizing parasitic resistance and capacitance typically associated with wire bonding. On-chip drivers replace external components, reducing LED failures and improving display system stability by leveraging the extended lifespan of GaN LED chips. This integration supports more compact, multifunctional systems, aligning with the trend towards miniaturization and the multifunctional benefits of integrated circuits (ICs). With the increasing demand for microdisplays, growth integration is expected to gain preference. However, InGaN/GaN LEDs may experience reduced internal quantum efficiency with red-shift spectra, leading to uneven subpixel emissions in full-color displays. Managing material growth compatibility and device processing remains a primary challenge, and wafer size limitations restrict the construction of large-scale micro-LED displays. Effective thermal budget management and thoughtful device structure design are crucial. Despite these benefits, challenges persist in achieving full-color integration and driver compatibility with this technology [8].

## PHOTONIC CRYSTAL BAND ENGINEERING FOR EXCITONIC MICRO-LEDs

**Figure 5.**
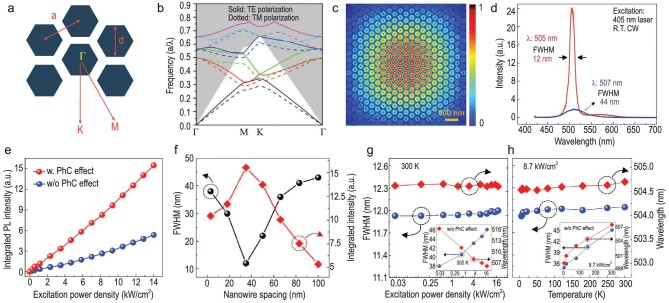
Simulation characteristics of (a) photonic crystals formation, with parameters such as lattice constant *a*, nanowire lateral size *d*, and reciprocal lattice vectors. (b) Calculated photonic band structure of the 2D hexagonal array of nanowires. (c) Electric field profile of the band edge mode (using the 3D finite-difference time-domain method, at a wavelength (λ) of 505 nm). (d) PL spectra of space-controlled dot-in-nanowire InGaN/AlGaN photonic crystals (red) compared to traditional InGaN/AlGaN nanowire (blue) measured at room-temperature. (e) Light intensity variations correspond to excitation power density at room-temperature. (f) Relationship between InGaN photonic crystal nanowire spacing, integrated luminescence intensity, and full width at half maximum (FWHM); performance characteristics between the emission peak and spectral linewidth in InGaN photonic crystal exhibit variations with (g) excitation power and (h) temperature, respectively (inset: without photonic crystal effect). Reproduced with permission from Ref. [24], Copyright 2017 WILEY-VCH Verlag GmbH & Co. KGaA, Weinheim.

Apart from the active medium, factors such as the optical density of states within the active region of a semiconductor light emitter are crucial in determining emission characteristics, known as the photonic crystal effect. Furthermore, in the dynamic setting of photonic crystal configurations, where the spacing between photonic nanowires varies while the nanowire diameter remains consistent, an intriguing interplay emerges. This interaction results in notable shifts in emission wavelength and spectral linewidth, enhancing the complexity of photonic crystal dynamics. Whether spacing decreases or increases, it wields control over the emission wavelength while tweaking the spectral linewidth, indicative of fluctuations in the coupling between quantum well spontaneous emission and band edge mode—affecting wavelength intensity. This distinct feature minimizes the adverse effects of Shockley–Read–Hall recombination, mitigating enduring electron-hole interactions. Advanced photonic crystals, tailored specifically for optimizing light extraction from the active region, utilize the Purcell effect within optical micro-cavities to shorten radiative lifetimes. This approach boosts internal quantum efficiency, particularly evident in InGaN nanowires, where exciton oscillator strength sees a substantial enhancement [104]. Typically, Ho Ra *et al*. [24] have revealed that InGaN/AlGaN dot-in-wire photonic crystals exhibit strong emission at ∼505 nm with spectral linewidths (FWHM) around 12 nm, as depicted in Fig. 5d. Additionally, corresponding simulation studies on photonic crystal formation, including the calculated photonic band structure of the 2D hexagonal array of nanowires and the electric field profile of the band edge mode (using the 3D finite-difference time-domain method at a wavelength of 505 nm), are depicted in Fig. 5d. By creating a bandgap aligned with the emission wavelength, these crystals facilitate selective out-coupling of light, reducing internal reflections, and boosting overall device efficiency. As injection current rises, excitonic µLEDs exhibit a swift increase in both EQE and device output power, surpassing conventional InGaN quantum well (QW) µLED performance (see Fig. 5e). Efficient surface strain relaxation through selective area epitaxy minimizes defects and dislocations, allowing precise control over photonic crystal morphology. Stable luminescent emission is achieved over a wide temperature range due to the strong Purcell effect. Moreover, their research has demonstrated that achieving optimal spectral linewidth and integrated intensity in the epitaxial structure depends on a nanowire spacing of 35 nm (see Fig. 5f). Utilizing this feature in InGaN/AlGaN dot-in-wire photonic crystal LEDs has resulted in almost three orders of magnitude higher integrated intensity compared to excitation power density. This underscores the importance of optimizing nanowire spacing, as inadequate configurations lead to reduced coupling between quantum dot spontaneous emission and the band edge mode, as shown in Fig. 5g. This robustness is attributed to the strong Purcell effect, enabled by the efficient coupling of spontaneous emission to highly stable and scalable band-edge modes. Furthermore, InGaN/AlGaN dot-in-wire photonic crystal LEDs demonstrate stable luminescent emission within a temperature range of 5–300 K. This stability persisted even under varying excitation densities from 29 W cm^−2^ to 17.5 kW cm^−2^, as illustrated in Fig. 5h.

Recently, Pandey *et al*. [23] explored the influence of excitons on the performance of µLEDs. They employed arrays with varying diameters (125 and 165 nm) but with the same lattice constant. The investigation revealed that, despite the smaller nanowire (NW) diameter of 125 nm, the device exhibited rectifying behavior and minimal reverse bias leakage. This remarkable behavior resulted in an exceptionally high peak EQE of 25.2%. In comparison, the device with a 165 nm diameter demonstrated a distinct excitonic effect, as evidenced by the well-defined p–n junction formation observed in the current-voltage (J-V) characteristics. In contrast, c-plane QW µLEDs, the excitonic effect is weakened due to strong QCSE and high plasma damage caused by mesa etching, which makes it difficult to observe the effect at low injection currents. At high injection currents, the presence of indium-rich nanoclusters becomes saturated, and most of the emission arises from the diffuse c-plane InGaN through free electron-hole recombination, rather than excitonic emission. Furthermore, EL characteristics reveal relative EQE for the lower-energy emission peak, at ∼515 nm, in Device A (attributed to excitonic emission), and the higher-energy emission peak around 490 nm. Band-to-band recombination from the central region of the NW (c-plane quantum disks) takes over, resulting in higher energy emission at ∼475–490 nm. In contrast, a prevalence of free electron-hole recombination occurs in Device B, resulting in the EL emission peak energy being observed beyond the maximum EQE of 4.1%. This significant difference in emission types at the same injection rate, leads to an unparalleled level of efficiency, previously unattainable for µLEDs. On the contrary, Device B does not display a substantial excitonic effect, resulting in the low-energy emission remaining negligible across the entire range of measured currents. Instead, it can be traced back to the excitonic emission originating from the strain-relaxed semi-polar quantum disks formed in dislocation-free NW arrays.

## PHOTONIC CRYSTAL BAND ENGINEERING FOR SURFACE EMITTING LASER (PC-SEL)

**Figure 6.**
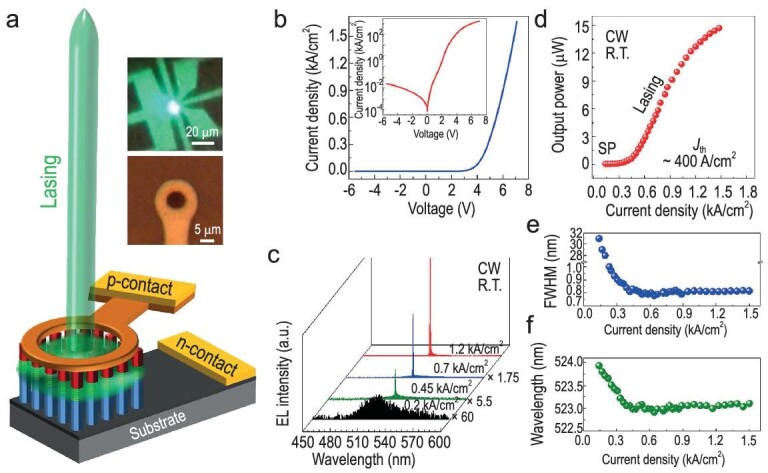
(a) Schematic demonstration of the fabricated NCSEL device. Inset: Optical microscopy image of the device after metallic contact grids and EL image of the green lasing. (b) I–V studies of the NCSEL device. Inset: The I–V curve on a semi-log scale. (c) EL spectra corresponds to various carrier injection rates under CW bias at room temperature. (d) Injection current corresponds to output power. (e) Injection current corresponds to spectral linewidth (FWHM). (f) Peak wavelength position measured under different injection current densities. Reproduced with permission from Ref. [105], Copyright 2020 AAAS, under a Creative Commons Attribution Non-Commercial License 4.0 (CC BY-NC).

Addressing the demand for high-quality Dielectric Bragg Reflectors (DBRs) in vertical-cavity surface-emitting lasers (VCSELs) poses a significant challenge due to the intricate lattice mismatch between III-nitride layers and marginal differences in dielectric constants [105,106]. The pronounced 2.5% mismatch between GaN and AlN, along with an ∼11% disparity between GaN and InN, poses a critical challenge [105,107]. Moreover, contending with the formidable task of achieving adept p-type conduction [108] leads to GaN-based DBRs marked by heightened electrical resistivity and significant instances of defects and dislocations [109,110]. The formidable impact of the polarization field and the resulting QCSE in traditional c-plane GaN devices obstructs radiative recombination, resulting in heightened thresholds and operational instability [111]. In response to these challenges, there has been a noteworthy shift towards the development of Vertical Cavity Surface Emitting Lasers (VCSELs) utilizing Gallium Nitride (GaN). This entails the integration of AlInN/GaN Distributed Bragg Reflectors (DBRs) [112] or a dual-dielectric DBR concept [113]. Adding to the complexity, m-plane GaN devices introduce a unique challenge, particularly due to a significantly elevated threshold current density (J_th_ >10 kA cm^−2^) at room temperature, with a responsive emissive region from 400 to 460 nm spectrum [114]. Ra *et al*. [105] have shown the exploration of DBR-free electrically injected surface-emitting green lasers employing dislocation-free arrays of gallium nitride nanocrystals, which exploit photonic band edge modes. A typical InGaN nanocrystalline surface-emitting laser (NCSEL) comprises a 370 nm thick n-GaN, followed by InGaN MQD as the active region and 190 nm p-GaN, as illustrated in Fig. 6. As illustrated in Fig. 6b, the 3 × 3 µm µLED device, when subjected to a low injection current density of ∼200 A/cm^2^, manifests a broad emission spectrum centered at ∼524 nm, exhibiting a FWHM of ∼30 nm, which corresponds to spontaneous emission. Remarkably, a sharp lasing peak emerges at ∼523.1-nm wavelength with an increasing injection current, displaying a narrow linewidth of ∼0.8 nm at 1 kA/cm^−2^. Moreover, in the exploration where the spectral linewidth experienced a reduction from ∼30 to 0.8 nm upon reaching the threshold, a consistent emission at ∼523 nm was observed in EL studies, as shown in Fig. 6c. Remarkably, this cutting-edge design harnesses the intrinsic capabilities of photonic band edge modes, strategically formed within flawless dislocation-free arrays of gallium nitride nanocrystals. NCSEL unveils a unique array of InGaN/AlGaN nanocrystals, which, through the photonic band edge resonant effect, allows the formation of standing waves. This eliminates the necessity for bulky and resistive Distributed Bragg Reflectors (DBRs) and envelops the active region, effectively suppressing surface recombination. Operating at a wavelength of 523.1 nm, the device demonstrates a minimal threshold current density of ∼400 A/cm², ensuring robust performance at room temperature. In essence, NCSEL pioneers a revolutionary paradigm in laser diode design, showcasing heightened efficiency and stability through the fusion of sophisticated nanocrystal engineering and groundbreaking photonics.

Furthermore, by leveraging cutting-edge advancements in photonic crystals, significant strides have been made in fine-tuning the spreading ratio enabled by the integration of InGaN tunnel junctions alongside photonic crystal nanowire capabilities. This integration has resulted in a notable reduction in power consumption by minimizing resistance. The breakthrough renders photonic crystals particularly well-suited for battery-dependent devices. This strategic integration overcomes the barriers posed by high resistance in p-GaN layers in conventional setups, thereby reducing the need for additional current spreading layers such as indium tin oxide (ITO) or flip-chips. Anticipated benefits of this fusion include enhanced contact current spreading, internal quantum efficiency, and light extraction efficiency. Consequently, it sets the stage for the advancement of photonic crystal-based DBR-free NCSEL with unprecedented performance capabilities. Notably, the electroluminescence spectra of four distinct photonic crystal lasers are scrutinized across a temperature spectrum spanning 12–375 K under 1.5 kA/cm², and reported by Ra *et al.* [26] which unveil remarkable constancy in emission wavelengths within this temperature domain, underscoring the resilience of the system under diverse thermal conditions. This precision allows the development of spatially extended band edge modes over a large defect-free photonic crystal area.

**Figure 7.**
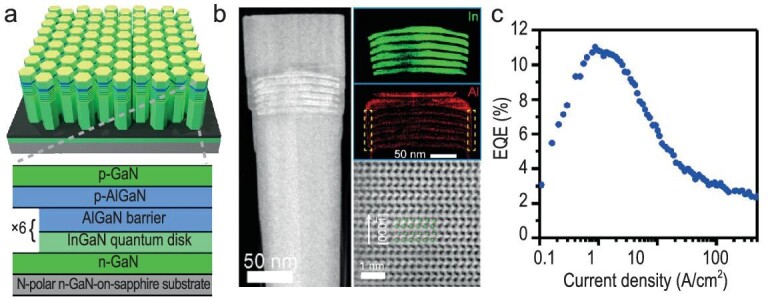
(a) Schematic diagram of n-polar n-GaN-InGaN/AlGaN-p-GaN nanowires, depicting the p-i-n heterostructure, with a detailed segment highlighting the LED heterostructure; (b) presents a STEM-HAADF image of the corresponding n-GaN-InGaN/AlGaN-p-GaN nanowire, along with elemental mapping showcasing the distribution of In and Al within the region, and an annular bright-field image revealing the atomic stack order. In the latter image, green balls denote Ga atoms, while red balls denote N atoms. (c) Variation of external quantum efficiency (EQE) with current density for the n-GaN- InGaN/AlGaN-p-GaN nanowire. Reproduced with permission from Ref. [71], Copyright 2022 Chinese Laser Press.

In pursuit of optimal efficiency in longer emissions, X. Liu *et al*. [104] reported the fabrication of strain-relaxed n-polar GaN-based InGaN nanowires on sapphire using SAG-MBE. A six-period InGaN quantum disk/AlGaN barrier active region with dimensions of 750 nm was grown over a base n-GaN segment. This was followed by the deposition of a p-type AlGaN electron-blocking layer and a p-GaN contact layer, as illustrated in Fig. 7. These facets emerged along semi-polar planes, displaying diverse indium compositions and contributing to a complex geometry in the active region, to mitigate polarization effects, as shown in Fig. 7b [23]. The advantages of strain relaxation and well-defined InGaN active regions on these facets facilitated charge carrier localization, enhancing excitonic recombination and potentially boosting the peak EQE by 11% on the wafer at a low carrier injection of 0.83 A/cm^−2^. Further investigation disclosed that the IQE at room temperature reached ∼60% for a green-emitting nanowire µLEDs operating at an injection current density of ∼1 A/cm^−^². The study utilized exciton oscillator strength and nanoscale quantum confinement for controlled emission, achieving highly efficient excitonic green submicron-scale nanowire LEDs within quantum-confined nanostructures. For instance, a notable peak EQE of 25.2% at a low injection current rate was achieved using the photonic crystal effect with green-emitting nanowires in the fabrication of µLEDs with InGaN nanowires [23]. They effectively utilized the interplay between exciton oscillator strength and quantum confinement. This spectral antagonist instigated the disassociation of excitons, leading to the untethering and subsequent recombination of free carriers—electrons and holes—away from the core of the nanowire, estranged from the comforting hold of its facets [6]. This unsettling phenomenon inflicted destruction upon efficiency, resulting in a precipitous decline above the 0.3 A/cm² threshold.

**Figure 8.**
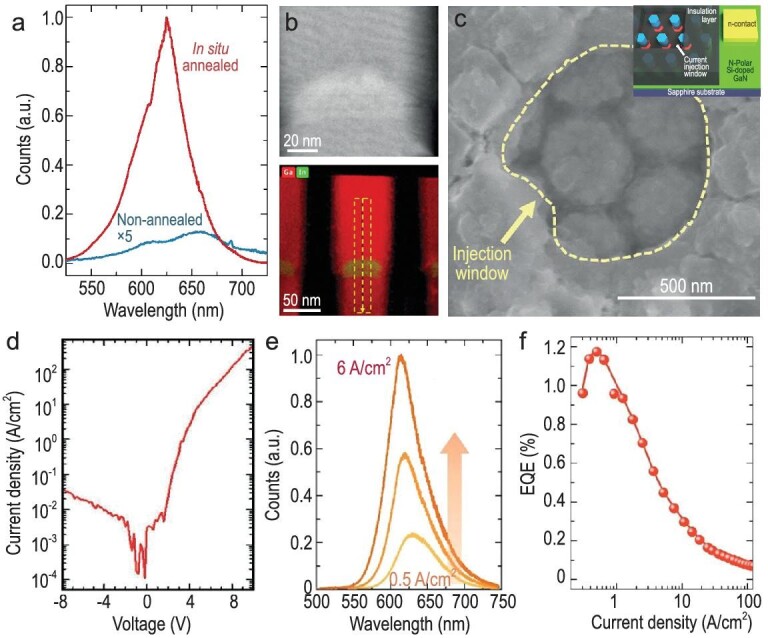
(a) PL spectra of InGaN/GaN heterostructure nanowires measured at room temperature for the samples non-annealed (blue) and *in situ* annealed (red); (b) presents a STEM-HAADF image of the corresponding InGaN/GaN nanowire (top), along with elemental mapping indicating the distribution of indium (In) and gallium (Ga) (bottom). (c) SEM image of submicron-scale device featuring an injection window highlighted by a yellow dashed curve. Inset: the scheme of the device corresponds to an InGaN/GaN µ-LED, showcasing the current injection window prior to the application of the p-metal contact layer. (d) J-V characteristics of µLEDs utilizing InGaN/GaN nanowires. (e) EL spectra corresponds to injection currents of µLEDs utilizing InGaN/GaN nanowires; (f) variation of EQE with current density for micro-LEDs employing InGaN/GaN nanowires. Reproduced with permission from Ref. [25], Copyright 2022 Chinese Laser Press.

Achieving high emission efficiency in the visible red region is a well-known challenge, primarily due to the difficulty of incorporating high indium content in III-nitrides. While smaller bandgaps of AlInGaP alloys are promising for efficient yellow-red emissions compared to III-nitride LEDs, AlGaInP-based LEDs face lower light emission efficiency despite achieving maximum (100%) IQE through lattice-matched hetero-epitaxial growth [115]. This inefficiency is attributed to high surface recombination velocities, particularly problematic in small-area devices where severe sidewall trapping effects occur in AlGaInP-based quantum wells (QWs) as chip sizes shrink, influenced by lower effective mass and larger carrier mobility [116]. Moreover, AlGaInP LEDs demonstrate poorer temperature stability and shorter wavelength emission tunability than InGaN counterparts, limiting their use in heat-sensitive applications. Conversely, InGaN offers significant advantages for long-wavelength red devices, covering the entire visible spectrum and enabling full-color RGB devices from a single material. InGaN-based LEDs benefit from superior temperature stability due to better quantum confinement of charge carriers, which enhances performance in various applications, especially in micro-LEDs smaller than 5 µm. Lower surface recombination velocities in III-nitrides further enhance suitability for small-area devices, where surface recombination is a critical factor. Following the notable success of submicron N-polar InGaN green-emitting LEDs, endeavors have shifted towards enhancing their capabilities to encompass extended wavelengths within the red spectrum—a pivotal initiative for the progress of display technologies. The foremost strategy in enhancing efficiency involves minimizing the QCSE, achieved through an increase in the thickness of the InGaN layer. Generally, annealing processes are pivotal for enhancing the structural integrity of materials. In this broader context, they make a substantial contribution to elevating the overall quality of the crystal, consequently positively influencing the emission characteristics of the active region. This strategic annealing approach, executed at a temperature slightly higher than the growth temperature, aligns with the overarching objective of improving crystal quality by proficiently diminishing the density of state defects [117]. By employing this precise annealing strategy, the refinement process becomes integral to the optimization of crystal properties, ensuring a more robust and defect-reduced material structure. Recently, Pandey *et al*. [25] demonstrated a significant tenfold enhancement in photoluminescence emission observed from the active region of the device through the utilization of *in-situ* annealing at a temperature 50°C higher than the actual growth condition (see Fig. 8a). The *in-situ* annealing sample, characterized by thicker and inhomogeneous InGaN layers, exhibited composition pulling and In distribution, as revealed through STEM analysis (see Fig. 8b). The fabricated LED device, with dimensions of 750 × 750 nm using these InGaN NWs, displayed nearly leakage-free behavior with a sharp turn-on at ∼2.5 V, as indicated by the J-V characteristics Fig. 8d. Furthermore, current-dependent EL studies demonstrated red emission characteristics with a peak wavelength around ∼620 nm at lower injection levels. As the injection rate increased, the EL emission peak underwent a shift towards the lower wavelength region, referred to as a blue-shift due to QCSE (see Fig. 8e). The maximum EQE of the device was recorded ∼1.2% at 0.5 A/cm^−2^ (see Fig. 8f).

## ADVANCEMENTS ON INTEGRATION WITH InGaN QD MICRO-LEDs: UNVEILING NEW FRONTIERS

**Figure 9.**
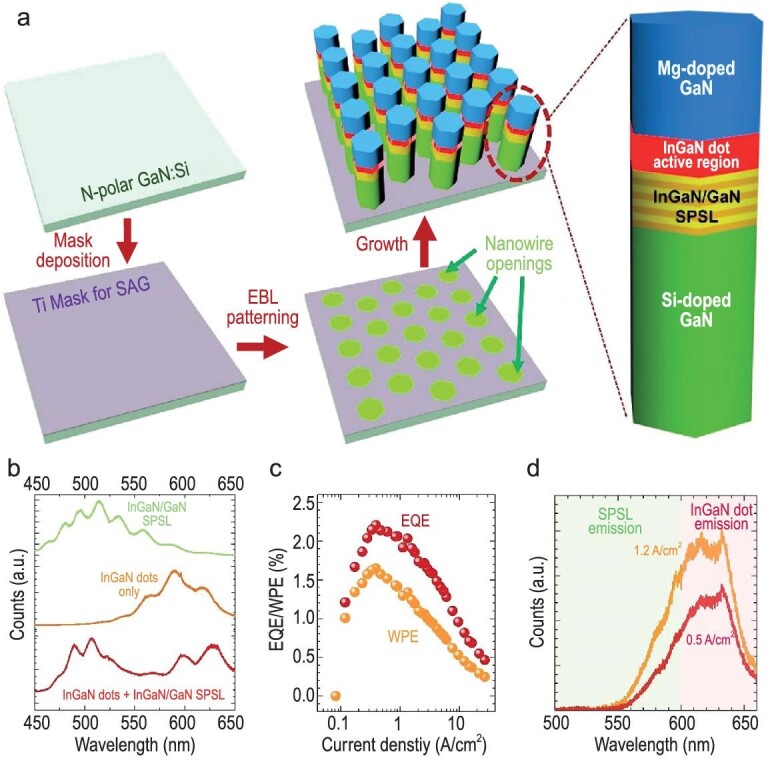
(a) Schematic representation of SAG patterning followed by growth structure of an epitaxial N-polar InGaN/GaN heterostructure nanowire array. (b) PL spectra of such InGaN/GaN SPSL, InGaN QDs, and InGaN/GaN SPSL with InGaN QD, respectively. (c) Current-dependent EQE and WPE of a submicron red LED with an InGaN/GaN SPSL incorporated lower in the active region. (d) EL spectra of the InGaN/GaN µLED measured at different injection currents. Reproduced with permission from Ref. [73], Copyright 2022 Chinese Laser Press.

Low-temperature crystal growth holds potential for meeting high In content requirements in red emissions but may hinder adatom mobility, leading to increased defects and degraded surface morphology. Additionally, lattice mismatch between the InGaN active region and the GaN substrate can cause extensive defect formation and high compressive strain on the InGaN layer, leading to the QCSE and reduced radiative recombination efficiency [118]. Nanoscale engineering techniques like porous GaN templates, V pits, and nanostructures have been explored to address these challenges [119]. A recent breakthrough involves the integration of high In-content InGaN QDs within a strain-relaxed short-period superlattice (SPSL) configured in a nanowire geometry, as reported by Pandey *et al*. [73]. Figure 9a illustrates the scheme of InGaN QD embedded onto the SPSL structure. The strain relaxation benefits of the SPSL layer can be further enhanced by incorporating QD nanostructures, which offer an increased surface area to volume ratio, facilitating more efficient red emission and overall improved performance. And also, this approach maintains the advantages of nanowire processing, such as avoiding a mesa etch step during µLED fabrication. The advancements of the InGaN/GaN-SPSL demonstrated the significant increment in emission characteristics such as peak emission at 630 nm with of peak EQE value of 2.2%, as shown in Fig. 9c.

Despite these strides, challenges remain, particularly concerning elevated turn-on voltages and reduced wall-plug efficiency (WPE) due to issues with p-GaN contact layers and the integration of electron-blocking layers and tunnel junctions within device heterostructures. Leveraging strain-relaxed InGaN QDs on SPSL nanowires has shown promising results, achieving substantial enhancement through optimized p-doping using Mg, resulting in an EQE of ∼8.3% and a WPE of ∼4.6% at a current density of 1 A/cm² for red-emitting sub-micrometer scale LEDs operating beyond 630 nm wavelengths [15]. This advancement improves Mg incorporation efficiency, mitigating defects associated with nitrogen vacancies and countering plasma damage on contacts, thereby significantly boosting device efficiency compared to lower doping levels. Overall, integrating strain-relaxed InGaN QDs within SPSL nanowires marks a notable stride towards higher-efficiency red-emitting micro-LEDs.

## MULTICOLOR NANOWIRE LEDs

**Figure 10.**
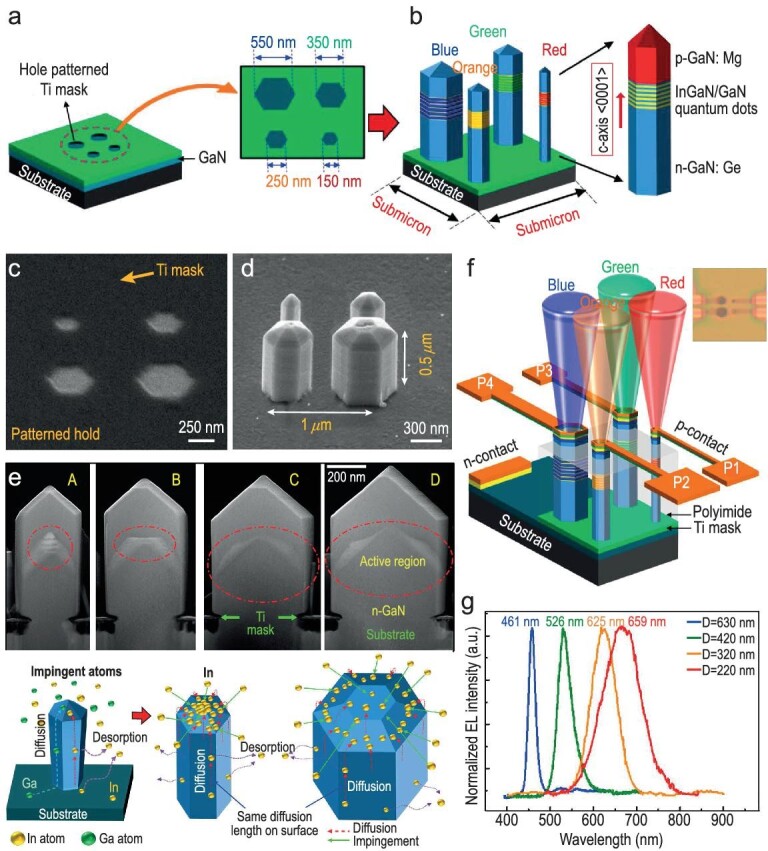
(a and b) Schematic illustration and (c) the corresponding SEM image of the patterning substrate for SAG; (d) showcases individual InGaN/GaN nanowires with varying diameters using SAG, as observed in the SEM image; (e) presents STEM-HAADF images of individual InGaN/GaN nanowires along the <1120> plane, with diameters labeled as (A) 320 nm, (B) 420 nm, (C) 500 nm, and (D) 595 nm, respectively, along with a schematic of the nanowire growth mechanism at the bottom; (f) provides a schematic representation of the device formation of on-chip monolithic multicolor single nanowire LED pixels (with a photograph of the corresponding device from a top-view perspective); (g) shows the electroluminescence (EL) spectra of the on-chip monolithic multicolor single nanowire LED. Reproduced with permission from Ref. [85], Copyright 2016 American Chemical Society.

On-chip monolithic multicolored InGaN nanowire µLEDs represent a state-of-the-art breakthrough in the field of µLED technology, however, it remains a daunting challenge. Achieving desirable emission necessitates precise adjustment of alloy compositions within various nanowire structures, a task that must be accomplished in a single growth or synthesis phase. Previous reports have demonstrated that controlled dimensional adjustments of InGaN nanowires using SAG significantly regulate the emission wavelength. This regulation is achieved by controlling the lateral surfaces of nanowires to regulate the In migration ratio into the InGaN nanostructure, even at consistent temperatures, resulting in controlled emission [21]. A pivotal aspect of these technological strides lies in the realization of full-color, tunable light sources, encompassing both µLEDs and lasers, utilizing addressable controlled-emissive individual nanowires integrated on a single substrate. Figure 10a–b illustrates the scheme of precisely controlled Ti mask patterns on an n-GaN template for SAG-MBE ranging from 80 nm to 1.9 µm, followed by the fabrication of InGaN nanowires onto the corresponding n-GaN openings, respectively. An SEM image of the SAG pattern mask and corresponding size-controlled grown nanowires is shown in Fig. 10c–d which consisted of epitaxial layers comprising ∼0.35 μm of GaN, five vertically aligned InGaN/GaN quantum dots, and a capping layer of ∼0.15 μm GaN, respectively. The nanowires that emerge display a nearly flawless hexagonal structure and are determined to possess Ga-polarity, as discerned from their terminating facets. Typically, in the case of small-diameter nanowires, high In-substituted quantum dots are positioned at the center of nanowires, which are vertically aligned along the c-axis. As the nanowire size increases, the position of In-substitution in quantum dots within the nanowire is reduced, becoming more dominant on the semi-polar planes. Figure 10f demonstrates the scheme of a single pixel multicolor nanowire device. As observed, electroluminescence characteristics using nanowire µLED devices show strong emission in the blue, green, orange, and red spectral wavelengths corresponding to the size of nanowires of 220, 320, 420, and 630 nm, respectively, as shown in Fig. 10g. Additionally, the nanowire geometry enhances light extraction, a key factor in achieving brighter and more energy-efficient displays. A remarkable characteristic of these multicolor InGaN nanowire µLEDs is their potential for integration into flexible and transparent substrates.

## InGaN NANOWIRE FULL-COLOR (WHITE) LEDs

**Figure 11.**
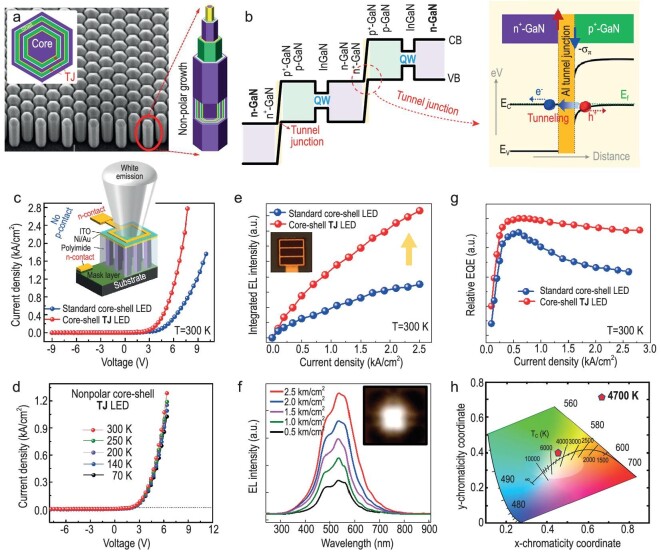
(a) SEM with schematic illustration of a nonpolar core–shell Al tunnel junction heterostructure. (b) Energy band diagram of a multiple-stacked core–shell tunnel junction structure with two Al tunnel junctions and InGaN quantum wells, (c) I–V studies of the multiple-stacked standard core–shell LED and multiple-stacked tunnel junction core–shell LED. Inset: device structure. (e) Light intensity versus current density measured at room temperature. (f) EL spectrum corresponding to injection current. (g) EQ vs. current density. (h) CIE chromaticity diagram. Reproduced with permission from Ref. [120], Copyright 2020 American Chemical Society.

To date, solid-state lighting has predominantly relied on GaN-based white LED technology, which employs phosphor-converted full-color emissions. However, this method faces energy losses due to the Stokes shift during wavelength down conversion of yellow/red from GaN blue or near-violet emissions, as well as complex packaging procedures exacerbating existing challenges. To address these issues, endeavors are underway to develop high-efficiency phosphor-free white LEDs by coupling blue/green and red emissions [120,121]. In this pursuit, attention has turned to InGaN-based LEDs, which utilize the coupling of RGB planar QWs for adjustable emission capabilities across the visible spectrum. Despite the potential of InGaN multiple quantum wells (MQWs) to emit polychromatic light, they encounter challenges with hole injection, primarily due to low mobility and the piezoelectric field. Additionally, high current injection levels are necessary, and lattice-mismatch strain issues at green and yellow emissions pose further complications. To address polarization-related issues, nonpolar structures are grown along the m-plane direction in GaN, resulting in improved electrical performance and light output power [122]. In a recent development, Ra *et al*. [120] reported on tunnel-junction-assisted n-GaN/Al metal/p-GaN polarization-enhanced nonpolar core-shell InGaN nanowire structures for phosphor-free white LEDs on Si. Figure 11 illustrates n^++^-GaN/Al/p^++^-GaN–Al tunnel junction, which exhibits an ohmic contact structure to n-GaN:Si doping layer and a quasi-ohmic contact to p-GaN:Mg doping layer at their interfaces. The presence of additional deep energy levels at the interface of the p^++^-GaN layer, grown with high Mg-doping, facilitates enhanced carrier transport through the trap-assisted tunneling effect. The presence of an Al interlayer between the p-GaN and n-GaN not only eliminates the necessity for polarization engineering but also results in an effective barrier width and inter-band conduction. Employing a nonpolar m-plane with an Al tunnel junction enhances luminescence efficiency, reduces the quantum confined Stark effect, improves current spreading, and enlarges the light extraction area. The nanowire structure aids in stress relaxation, reducing dislocations and contributing to the formation of high-quality Al and doping epitaxial layers on the GaN structure. The as-fabricated TJ core–shell LED presents distinctive EL traits, showcasing a peak EQE at ∼500 A/cm², while maintaining minimal efficiency droop as the current density escalates to 2700 A/cm². Moreover, the recorded CCT value at ∼4700 K is indicative of a remarkably steadfast white emission, coupled with an exceptionally efficient hole injection through an Al-TJ, and a remarkably stable carrier recombination within a nonpolar active region encapsulated in the core-shell structure. In a recent study by Wan *et al*. [123], it was demonstrated that a single-chip WLED incorporating self-assembled InGaN QDs achieved a Color Rendering Index (CRI) of 75. This was coupled with an exceptional modulation bandwidth of 150 MHz at a minimal current density of 72 A/cm⁻², along with a −3 dB modulation depth. These findings signify a remarkable advancement in solid-state lighting technology, offering the potential for brighter, more vibrant, and energy-efficient illumination across various settings.

## SUMMARY, PROSPECTS, AND RESEARCH OPPORTUNITIES OF InGaN NANOWIRES

Advancements in scalable InGaN nanowire µLEDs encompass improvements in emission efficiency, size reduction, color uniformity, loss mitigation, and precision, all crucial for commercial viability. Research into monolithic single/multi/full-color emission is set to propel the development of monolithic color µLED displays, advancing next-generation display technology. Key challenges include improving efficiencies at low current densities, enhancing green and red emitter performance, addressing surface recombination in small devices, optimizing viewing angle coupling efficiency, and engineering devices for seamless display assembly. Notably, enhancing III-nitride green and red LEDs is crucial for significant display advancements, especially for large-scale, high-resolution displays in applications like AR/VR integration. While the commercialization of multi/full-color µLED displays is inevitable, further optimization of growth and integration technology is essential. Swift resolution of challenges, coupled with advances in complementary photonic components, could lead to widespread adoption of III-nitride NWs, potentially displacing flexible integrated photonics on a large scale.

Such exploration involves smart lighting systems that utilize the dynamic control over color temperature and intensity provided by monolithic multicolor InGaN nanowire µLEDs, use for visible light communications [124,125] and photon sensing [126]. Additionally, the application of these µLEDs in healthcare settings holds promise for non-invasive diagnostics, therapeutic interventions, fluorescence spectroscopy, and advanced imaging techniques [127]. Their precise illumination could significantly improve diagnostics in equipment such as endoscopes, and wearable health monitoring devices and sensors [128]. Furthermore, the fast response times of µLEDs make them suitable for integration into Li-Fi communication systems, enabling high-speed data transmission through visible light [129] with potential uses in heads-up displays, cockpit instrumentation, and advanced communication systems due to their durability, low power consumption, and reliability [130]. Automotive displays stand to benefit from the incorporation of µLEDs in heads-up displays, dashboard lighting, and interior ambient lighting, contributing to both safety and aesthetics.

Exploring three-dimensional nanowire architectures for µLEDs becomes crucial to enhance light extraction efficiency, color purity, and overall device performance. Moreover, the combination of monolithic multicolor InGaN nanowire µLEDs with other optical technologies, such as micro-optoelectromechanical systems (MOEMS) or diffractive optics, presents opportunities for dynamic beam shaping, tunable spectral characteristics, and improved light manipulation. Integrating these µLEDs with sensors, like photodetectors or environmental sensors, can result in intelligent systems capable of responsive and adaptive behavior, particularly beneficial in applications requiring real-time data processing [131]. The pursuit of advanced substrates, including flexible and transparent materials, opens the door to the development of flexible and foldable µLED displays. Additionally, the integration of 2D materials like graphene or transition metal dichalcogenides, along with fine-tuning quantum well structures in InGaN nanowires, aims to improve efficiency, color purity, and overall reliability of these µLEDs [132]. This ongoing exploration and innovation across diverse domains showcase the versatility and potential impact of monolithic multicolor InGaN nanowire µLEDs beyond their initial focus on display technologies.
